# Prognostic function to estimate the probability of meaningful clinical improvement after surgery - Results of a prospective multicenter observational cohort study on patients with lumbar spinal stenosis

**DOI:** 10.1371/journal.pone.0207126

**Published:** 2018-11-08

**Authors:** Ulrike Held, Jakob M. Burgstaller, Maria M. Wertli, Giuseppe Pichierri, Sebastian Winklhofer, Florian Brunner, François Porchet, Mazda Farshad, Johann Steurer

**Affiliations:** 1 Horten Centre for Patient Oriented Research and Knowledge Transfer, University of Zurich, Zurich, Switzerland; 2 Epidemiology, Biostatistics and Prevention Institute, University of Zurich, Zurich, Switzerland; 3 Division of General Internal Medicine, Bern University Hospital, Bern University, Bern, Switzerland; 4 Department of Neuroradiology, University Hospital Zurich, Zurich, Switzerland; 5 Department of Physical Medicine and Rheumatology, Balgrist University Hospital, Zurich, Switzerland; 6 Department of Orthopedics and Neurosurgery, Spine Center, Schulthess Clinic, Zurich, Switzerland; 7 Spine Division, Balgrist University Hospital, Zurich, Switzerland; University of California San Francisco, UNITED STATES

## Abstract

**Background:**

Approximately two thirds of patients with lumbar spinal stenosis (LSS) who undergo surgical treatment benefit from the surgery. The objective of this study was to derive a prognostic probability function (PPF) to identify patients with a high probability of post-surgical improvement because there is currently no method available.

**Methods:**

In this multicenter, prospective, observational study, we collected data from eight medical centers in Switzerland in which patients underwent surgery for LSS. The endpoints were meaningful clinically important differences (MCID) in pain and disability one year after baseline. We developed a PPF named PROCESS (PostopeRative OutComE Spinal Stenosis), based on a large set of prognostic indicators extracted from the literature. The PPF was derived using data from a random subset of two thirds of the patients and validated in the remaining third. We addressed overfitting by shrinking the regression coefficients. The area under the ROC curve (AUC) and calibration determined the accuracy of the PPF.

**Results:**

In this study, 452 LSS patients received surgery. 73% of the 300 patients in the derivation subset reached an MCID in pain and 68% reached an MCID in disability. The corresponding values were 70% and 63% in the validation subset, respectively. In the derivation subsample, the AUC was 0.64 (95% CI 0.57 to 0.71) for of the PPF predicting MCID in pain and 0.71 (0.64 to 0.77) for MCID in disability, after shrinkage. The corresponding numbers were 0.62 (0.52 to 0.72) and 0.70 (0.60 to 0.79) in the validation subsample, and the PPF showed good calibration.

**Conclusions:**

Surgical treatment for patients with lumbar spinal stenosis is being performed with increasing frequency. PROCESS is conditional on the individual pattern of preoperatively available prognostic indicators, and may be helpful for clinicians in counselling patients and in guiding the discussion on individual treatment decision in the era of personalized medicine.

## Introduction

Decompression surgery is a treatment option for patients with lumbar spinal stenosis. Surgical treatment is recommended for patients with moderate or severe clinical manifestations and no meaningful improvement following conservative treatment, such as physiotherapy and/or epidural steroid injections. Surveys among surgeons using standardized clinical cases revealed a lack of consensus among clinical experts on the indications for surgery [1], and the wide variation in surgical rates among hospital referral regions in the USA may be explained by this lack of consensus [2].

There is broad agreement that decompressive surgery offers an advantage for about two thirds of patients within the first four years after surgery compared to non-surgical treatment [3–6], while one third of patients report no meaningful improvement after surgery [4, 5]. This difference in outcome declines between surgical and non-surgical treatment five to ten years after surgery [7–9]. A tool for identifying patients with a high probability of post-surgical improvement would be valuable for patients and physicians in decision-making regarding surgery, and could reduce the rate of unnecessary operations.

The aim of this study was to develop a PPF for estimating the likelihood of post-surgical improvement in patients with lumbar spinal stenosis at one year follow-up, conditional on a set of prognostic indicators measured at baseline.

## Materials and methods

The Lumbar Stenosis Outcome Study (LSOS) is a prospective cohort study investigating the effectiveness of various treatment options in patients with symptomatic lumbar spinal stenosis [10]. Participation in the study had no influence on the treatment of the patients, all treatment decisions were left to the patient and physician. 841 patients were recruited from December 2010 to December 2015, and were followed-up for three years.

The present study is reported according to STROBE (Statement for reporting cohort studies) guidelines [11] and TRIPOD (Statement for studies reporting clinical prediction models) guidelines [12] (S1 and S2 Files).

### Inclusion and exclusion criteria

The following characteristics were required for study eligibility: age ≥50 years, uni- or bilateral neurogenic claudication, verified stenosis of the lumbar spinal canal determined by magnetic resonance imaging (MRI), life expectancy ≥1 year, ability to give informed consent, availability for follow-up, and ability to complete questionnaires in the German language. We excluded patients with cauda equina syndrome requiring urgent surgery, current fracture, infection, significant deformity (>15° lumbar scoliosis) of the lumbar spine, current enrollment in another spine-related treatment study, and clinically relevant peripheral arterial disease (as confirmed by a vascular specialist in patients without palpable lower limb pulse).

### Eligibility criteria for inclusion in the analysis

All patients who underwent surgery within six months of enrollment and with 12 months of follow-up data were included in the analysis.

### Surgical procedures and radiological classification

The surgery consisted of a standard open posterior lumbar laminotomy with or without instrumentation of the affected level(s). The surgeon’s discretion determined the decision to proceed with a laminotomy using unilateral technique, to decompress the contralateral recess, or to take a midline approach with bilateral laminotomy. Fusion surgery included implantation of pedicle screws with rods, plus intersomatic fusion and cage(s) at the affected level(s), in addition to decompression surgery. Additional fusion, single or multi-level decompression, or the use of an operating microscope was based on the surgeon’s discretion. The procedures were done or supervised by senior neuro- or orthopedic surgeons with more than ten years of experience after board-certification, and each patient’s MRI was independently evaluated by two senior radiologists.

### Data collection and follow-up

Baseline data was taken from interviews and recorded by a study coordinator. All other questionnaires were self-administered by the patients themselves. Data were collected at baseline and after 12 months.

### Outcome measures

*Spinal Stenosis Measure (SSM)*: The SSM is an instrument specifically developed and validated for spinal stenosis patients by Stucki and colleagues [13], and both measures symptom severity and quantifies disability in patients with lumbar spinal stenosis. The SSM is recommended by the North American Spine Society (NASS) and is used in many different studies of lumbar spinal stenosis [14–17]. Scores range from 1–5 and 1–4 (best-worst) for SSM symptoms score and SSM function score. A minimal clinically important difference (MCID) in SSM symptoms score is defined by an improvement of 0.48 points from baseline to 12 months, and an improvement of 0.52 defines an MCID in SSM function score [18].

### Development of a PPF and choice of prognostic indicators

A PPF derives the probability of a future event based on a set of prognostic indicators defined at a specified point in time [19]. In this application, the future event was MCID one year after baseline in SSM symptoms score or SSM function score. We studied the literature to generate a list of prognostic indicators [20–23]. The dichotomous and continuous parameters measured at baseline and available in the LSOS database were included in the PROCESS (PostopeRative OutComE Spinal Stenosis) PPF. The list of prognostic indicators is summarized in Table 1.

**Table 1:** List of 21 prognostic indicators used in the prognostic probability function for meaningful clinically important difference (MCID) in SSM symptoms score and SSM function score one year after baseline.

Prognostic indicator	
*Dichotomous*	*Unfavorable*
Age	≥75 years [21]
Gender	female
BMI	≥30 kg/m^2^ [24]
Current smoker	yes
Civil status	living alone, or single/divorced/widowed and living in a nursing/residential home
Formal education	compulsory school only
Coxarthrosis or gonarthrosis	yes
Coronary heart disease or heart insufficiency	yes
Asthma or COPD	yes
Parkinson’s disease or peripheral neuropathy	yes
Walking ability	being able to walk only up to 200 m
Low back pain	yes
Duration of symptoms	≥6 months [25]
Preoperative analgesic use within 3 months before baseline	yes
Previous lumbar surgery	yes
Number of decompressed levels	>1 level
Radiological parameters	Antero-posterior diameter of dural sac (APD) >6 mm or cross sectional area >70 mm^2^ [22]
Depression (on HADS depression scale)	≥8 points [26]
*Continuous*	*Range*
Quality of life (EQ5D-3L scale)	0 (worst)– 100 (best)
Baseline SSM symptoms score	1 (best)– 5 (worst)
Baseline SSM function score	1 (best)– 4 (worst)

BMI = body mass index; COPD = chronic obstructive pulmonary disease; HADS = Hospital Anxiety and Depression Scale; SSM = Spinal Stenosis Measure

### Patient scenarios

The PPF’s usability in terms of pain and disability was also assessed by characterizing two scenarios, one with a very favorable constellation of prognostic indicators, and the second with a disadvantageous constellation of prognostic indicators. We calculated the estimated probabilities of MCID in these two scenarios.

#### Scenario 1 (favorable)

Male patient, all favorable prognostic indicators present (Table 1), age <75 years, BMI <30 kg/m^2^, non-smoking, not living alone or single/divorced/widowed and living in a nursing/residential home, no low education, no cox- or gonarthrosis, coronary heart disease or heart insufficiency, no asthma or chronic obstructive pulmonary disease (COPD), no Parkinson’s disease or peripheral neuropathy, able to walk more than 200 m, no low back pain, duration of symptoms <6 months, analgesic use within 3 months before baseline, no previous lumbar surgery, surgery on one single lumbar spinal level, narrowing of the cross sectional area (≤70 mm^2^) or the diameter of the dural sac (≤6 mm), no depression, average quality of life score of 50 points, baseline SSM symptoms and function scores both 3.5.

#### Scenario 2 (unfavorable)

Female patient, all favorable prognostic indicators absent (Table 1), average quality of life score of 50, baseline SSM symptoms and function scores both 2.

### Statistical methods

Thorough development and validation of a PPF is important [27]. For that reason, patients were randomly split once into a *derivation subsample* (2/3 of the patients) for development of the PPF and a *validation subsample* (1/3 of the patients) for validation of the function, in order to determine the validity of results for new patients [28]. Descriptive statistics included median and interquartile ranges for continuous variables, and counts and percentages of total for categorical variables. Corresponding Wilcoxon and chi-squared tests were used to compare the two subsamples.

There was missing data for some of the patients for some of the prognostic indicators. These were filled using 10-fold multiple imputation based on chained equations [29], retaining the information about the derivation and validation subsamples.

The two binary outcome variables, MCID in SSM symptoms and SSM function were addressed with logistic regression models fitted to each outcome in each of the ten imputed derivation subsamples including all 21 prognostic indicators.

PPF models with a large number of prognostic indicators tend to describe optimally the data under study, but predictions for *new* subjects will perform less well. To address this phenomenon, called overfitting, the regression coefficients of the PPF can be shrinked towards zero by multiplying with a global shrinkage factor. E.g., a coefficient of 0.8 becomes 0.72 (= 0.8*0.9) if the shrinkage factor was 0.9. We derived a global shrinkage factor for the estimated regression coefficients using the *dfbeta*-method [30]. A global shrinkage factor for the model addressing MCID in SSM symptoms and a global shrinkage factor for MCID in SSM function was calculated in each of the ten imputed derivation subsamples and then averaged, resulting in one global shrinkage factor for each outcome.

Pooled regression coefficients were calculated from the ten derivation subsamples following Rubin’s rule [31], and the two global shrinkage factors were applied. Original and shrunken regression coefficients are summarized as odds ratios with 95% confidence intervals (CI). The pooled and shrunken regression coefficients were applied to each of the ten multiply imputed validation subsamples, resulting in predictions of the probability for MCID in SSM symptoms and SSM function scores.

After the derivation of a PPF, one would like to know how well the predicted probability for MCID (continuous between 0 and 1) corresponds to the actual observed MCID-status (0 or 1) in SSM symptoms and SSM function. This can be measured with the discriminative ability of the PPF, as well as with its calibration. A **r**eceiver **o**perating **c**haracteristic (ROC) plot displays the true positive rate against the false positive rate for consecutive cut-offs for the predicted probability. The **a**rea **u**nder this ROC **c**urve (AUC) with 95% CI is calculated to assess the discriminative ability of the PPF. Calibration is another important property of a probability function, and it measures the agreement between observed outcomes and predictions. We used calibration plots, for 10-fold imputed derivation and validation subsamples.

All analyses were conducted using R for Windows [32], using the packages *dplyr*, *MASS*, *mice*, *mitools*, *openxlsx*, *PresenceAbsence*, *pROC*, *rms*, *rpart*, *shrink*, and *tableone*. Our work was conducted following the concept of reproducible research and the R-code is available upon request [33].

### Sample size

The sample size was calculated for the development of a PPF for patients undergoing spine surgery, one year after surgery [10]. For sample size calculation, we anticipated that 60% of the included 841 patients (= 505 patients) with verified diagnosis would undergo surgery. Actually, 543 patients underwent surgery during the follow-up, 498 of these within the first six months after baseline and 452 of these had a follow-up of at least 12 months.

We anticipated that two thirds of the patients would show a clinically relevant improvement one year after surgery. Based these assumptions, the number of prognostic indicators in the logistic regression model in the derivation set may be up to 20 following the rule of 10 outcome events per predictor variable (EPV). According to Vittinghoff and McCulloch [34], this rule can be relaxed to 5 to 9 EPVs allowing for up to 22 prognostic indicators if 9 EPVs are taken as threshold.

### Ethical approval

This multi-centre cohort study was conducted in compliance with all international laws and regulations as well as any applicable guidelines. Written informed consent to participate in the study has been obtained from participants. The study was approved by the independent Ethics Committee of the Canton Zurich (KEK-ZH-NR: 2010-0395/0).

## Results

### Patient characteristics

**Fig 1:**
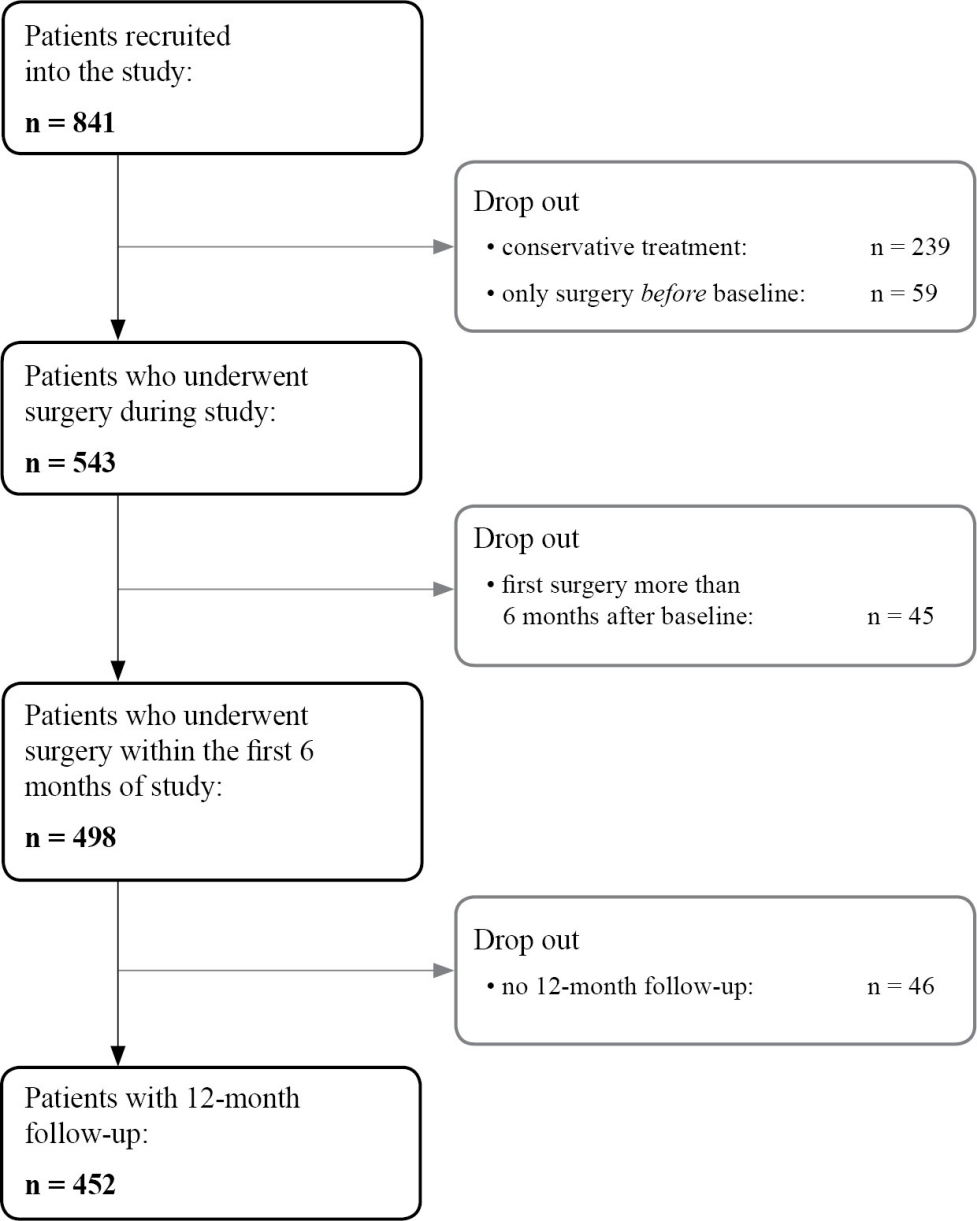
Patient flow chart.

Four hundred and fifty-two patients who received surgery for lumbar spinal stenosis within six months of baseline had 12-month follow-up data (Fig 1). 300 were randomly selected for the derivation subsample, and the remaining 152 patients were allocated to the validation subsample. 141 patients in the derivation subsample (47%) were 75 years or older, while 73 (48%) of patients in the validation subsample were in the same age range. 51% and 52%, respectively, were female. Details for all prognostic indicators are shown in Table 2. There were no differences between the subsamples across all variables.

**Table 2:** Preoperative baseline characteristics.

	Derivation data set	Validation data set	p-value
	N = 300	N = 152	
Age ≥75 years, No. (%)	141 (47.0)	73 (48.0)	0.915
Female gender, No. (%)	152 (50.7)	79 (52.0)	0.870
BMI ≥30 kg/m^2^, No. (%)	83 (27.7)	47 (30.9)	0.540
Current smoker, No. (%)	50 (16.7)	24 (15.9)	0.929
Living alone, or single/divorced/widowed and living in nursing home, No. (%)	95 (31.7)	56 (36.8)	0.319
Compulsory school only, No. (%)	70 (23.5)	45 (29.6)	0.196
Coxarthrosis or gonarthrosis, No. (%)	105 (38.0)	54 (36.7)	0.873
Coronary heart disease or heart insufficiency, No. (%)	19 (6.9)	10 (6.8)	1
Asthma or COPD, No. (%)	28 (10.0)	19 (12.8)	0.465
Parkinson’s disease or peripheral neuropathy, No. (%)	7 (2.5)	6 (4.1)	0.552
Being able to walk only up to 200m, No. (%)	209 (69.9)	99 (65.1)	0.357
Low back pain, No. (%)	263 (88.0)	133 (88.1)	1
Duration of symptoms ≥6 months, No. (%)	182 (61.1)	87 (58.0)	0.600
Preoperative analgesic use within 3 months before baseline, No. (%)	242 (80.9)	117 (78.5)	0.633
Previous lumbar surgery, No. (%)	27 (9.0)	22 (14.5)	0.108
More than one decompressed level, No. (%)	181 (60.5)	82 (55.8)	0.392
Diameter of the dural sac (APD) >6 mm or cross sectional area >70 mm^2^ (%), No. (%)	52 (17.3)	34 (22.4)	0.245
Depression on HADS scale ≥8, No. (%)	56 (18.7)	26 (17.2)	0.793
Quality of life on EQ5D actual health status, median [IQR]	64.0 [40.0, 80.0]	63.5 [40.0, 80.0]	0.818
Baseline SSM symptoms score, median [IQR]	3.1 [2.7, 3.6]	3.1 [2.9, 3.5]	0.912
Baseline SSM function score, median [IQR]	2.2 [1.8, 2.8]	2.2 [1.8, 2.8]	0.808

BMI = body mass index; COPD = chronic obstructive pulmonary disease; HADS = Hospital Anxiety and Depression Scale; SSM = Spinal Stenosis Measure

### MCID in SSM symptoms and SSM function scores

73% of the patients in the derivation subsample and 70% of patients in the validation subsample reached an MCID in SSM symptoms score after surgery. The percentages for MCID in SSM function score were 68% and 63%, respectively.

### Intra- and postoperative complications

Eighteen (6.0%) patients of the derivation subsample suffered an injury of the dura during surgery, the corresponding numbers were 9 (5.9%) in the validation subsample. No patient in the derivation subsample and three patients (2.0%) in the validation subsample experienced epidural venous bleeding.

In the derivation subsample, 5 (1.7%) patients had a wound infection, other complications (e.g., urosepsis, hemorrhage, wound healing deficit) were experienced by 25 (8.3%) patients. In the validation subsample, no wound infections were observed, and 15 (9.9%) patients had other complications. Twenty-two (7.3%) patients had a reoperation in the derivation subsample and 15 (9.9%) patients in the validation subsample.

### Multiple imputation and shrinkage factors

There were missing values for 18 of the prognostic indicators. The percentage of missing values varied between 0.2% in walking ability, and 6.4% for the coxarthrosis/gonarthrosis variable. Ten-fold multiple imputation was applied.

The continuous prognostic indicators baseline SSM symptoms score and SSM function score, and EQ-5D actual health status were entered in a linear as well as in a quadratic fashion and the residual plot was in favor of the linear effect for all three of them.

The resulting average global shrinkage factors were 0.47 for MCID in SSM symptoms score and 0.60 for MCID in SSM function score.

Pooled regression coefficients expressed as log odds ratios and 95% CIs are summarized in Table 3 (MCID in SSM symptoms score as outcome) and Table 4 (MCID in SSM function score as outcome). The shrinked regression coefficients are also displayed for each outcome. These were the final coefficients, and were used for calculating the probability of MCID in SSM symptoms score and SSM function score following surgery.

**Table 3:** PROCESS: Estimated coefficients and their 95% confidence intervals, p-values, and shrinked coefficients for the SSM symptoms score. The shrinkage factor was 0.47.

MCID in SSM symptoms	Coefficients = log odds ratios	Lower bound of 95% CI	Upper bound of 95% CI	p-value	Shrinked coefficients
(Intercept)	-1.212	-3.878	1.455	0.372	-0.565
Age	-0.460	-1.090	0.171	0.152	-0.214
Gender	0.108	-0.568	0.785	0.753	0.050
Body mass index	-0.443	-1.118	0.232	0.197	-0.207
Current smoker	-0.021	-0.853	0.811	0.960	-0.010
Civil status	-0.178	-0.874	0.519	0.616	-0.083
Formal education	-0.232	-0.924	0.460	0.510	-0.108
Coxarthrosis or gonarthrosis	-0.399	-1.041	0.243	0.221	-0.186
Coronary heart disease or heart insufficiency	-1.221	-2.348	-0.093	0.034	-0.569
Asthma or COPD	0.612	-0.609	1.833	0.324	0.285
Parkinson’s disease or peripheral neuropathy	-1.218	-3.115	0.679	0.206	-0.568
Walking ability	-0.189	-0.939	0.561	0.621	-0.088
Low back pain	-0.439	-1.405	0.528	0.372	-0.205
Duration of symptoms	0.004	-0.618	0.626	0.990	0.002
Preoperative analgesic use within 3 months before baseline	-0.070	-0.864	0.724	0.862	-0.033
Previous lumbar surgery	0.050	-0.958	1.057	0.923	0.023
Number of decompressed levels	-0.269	-0.882	0.344	0.389	-0.125
Antero-posterior diameter of dural sac (APD) >6 mm or cross sectional area >70 mm^2^	-0.694	-1.421	0.032	0.061	-0.324
Depression (on HADS depression scale)	-1.091	-1.872	-0.311	0.006	-0.509
Quality of life (EQ5D-3L scale)	0.001	-0.013	0.015	0.882	0
Baseline SSM symptoms score	1.396	0.761	2.032	<0.001	0.652
Baseline SSM function score	-0.174	-0.808	0.460	0.589	-0.081

CI = confidence interval; COPD = chronic obstructive pulmonary disease; HADS = Hospital Anxiety and Depression Scale; SSM = Spinal Stenosis Measure

**Table 4:** PROCESS: Estimated coefficients and their 95% confidence intervals, p-values, and shrinked coefficients for the SSM function score. The shrinkage factor was 0.60.

MCID in SSM function	Coefficients = log odds ratios	Lower bound of 95% CI	Upper bound of 95% CI	p-value	Shrinked coefficients
(Intercept)	-1.054	-3.692	1.584	0.432	-0.633
Age	-0.603	-1.233	0.028	0.061	-0.362
Gender	-0.002	-0.670	0.665	0.994	-0.001
Body mass index	-0.957	-1.634	-0.281	0.006	-0.574
Current smoker	0.467	-0.370	1.304	0.273	0.280
Civil status	0.592	-0.133	1.316	0.109	0.355
Formal education	0.113	-0.607	0.834	0.757	0.068
Coxarthrosis or gonarthrosis	-0.711	-1.364	-0.058	0.033	-0.427
Coronary heart disease or heart insufficiency	-0.742	-1.834	0.351	0.182	-0.445
Asthma or COPD	-0.722	-1.769	0.326	0.176	-0.433
Parkinson’s disease or peripheral neuropathy	-1.103	-2.930	0.725	0.234	-0.662
Walking ability	0.108	-0.676	0.892	0.786	0.065
Low back pain	-1.127	-2.111	-0.142	0.025	-0.676
Duration of symptoms	-0.046	-0.672	0.580	0.885	-0.028
Preoperative analgesic use within 3 months before baseline	-0.366	-1.168	0.435	0.369	-0.220
Previous lumbar surgery	-0.987	-1.951	-0.022	0.045	-0.592
Number of decompressed levels	-0.377	-1.001	0.247	0.235	-0.226
Antero-posterior diameter of dural sac (APD) >6 mm or cross sectional area >70 mm^2^	-0.571	-1.322	0.180	0.136	-0.343
Depression (on HADS depression scale)	-0.552	-1.350	0.247	0.175	-0.331
Quality of life (EQ5D-3L scale)	0.010	-0.004	0.025	0.169	0.006
Baseline SSM symptoms score	-0.239	-0.834	0.357	0.431	-0.143
Baseline SSM function score	2.038	1.312	2.764	<0.001	1.223

CI = confidence interval; COPD = chronic obstructive pulmonary disease; HADS = Hospital Anxiety and Depression Scale; SSM = Spinal Stenosis Measure

### Discriminative ability of PROCESS, calibration and validation

In the derivation subsample, the discriminative ability as measured with the AUC of the PPF was 0.64 (95% CI 0.57 to 0.71) for the MCID in SSM symptoms score after shrinkage. The AUC was 0.62 (0.52 to 0.72) in the validation subsample. The corresponding values were 0.71 (0.64 to 0.77) in the derivation subsample and 0.70 (0.60 to 0.79) in the validation subsample for the MCID in SSM function score.

**Fig 2:**
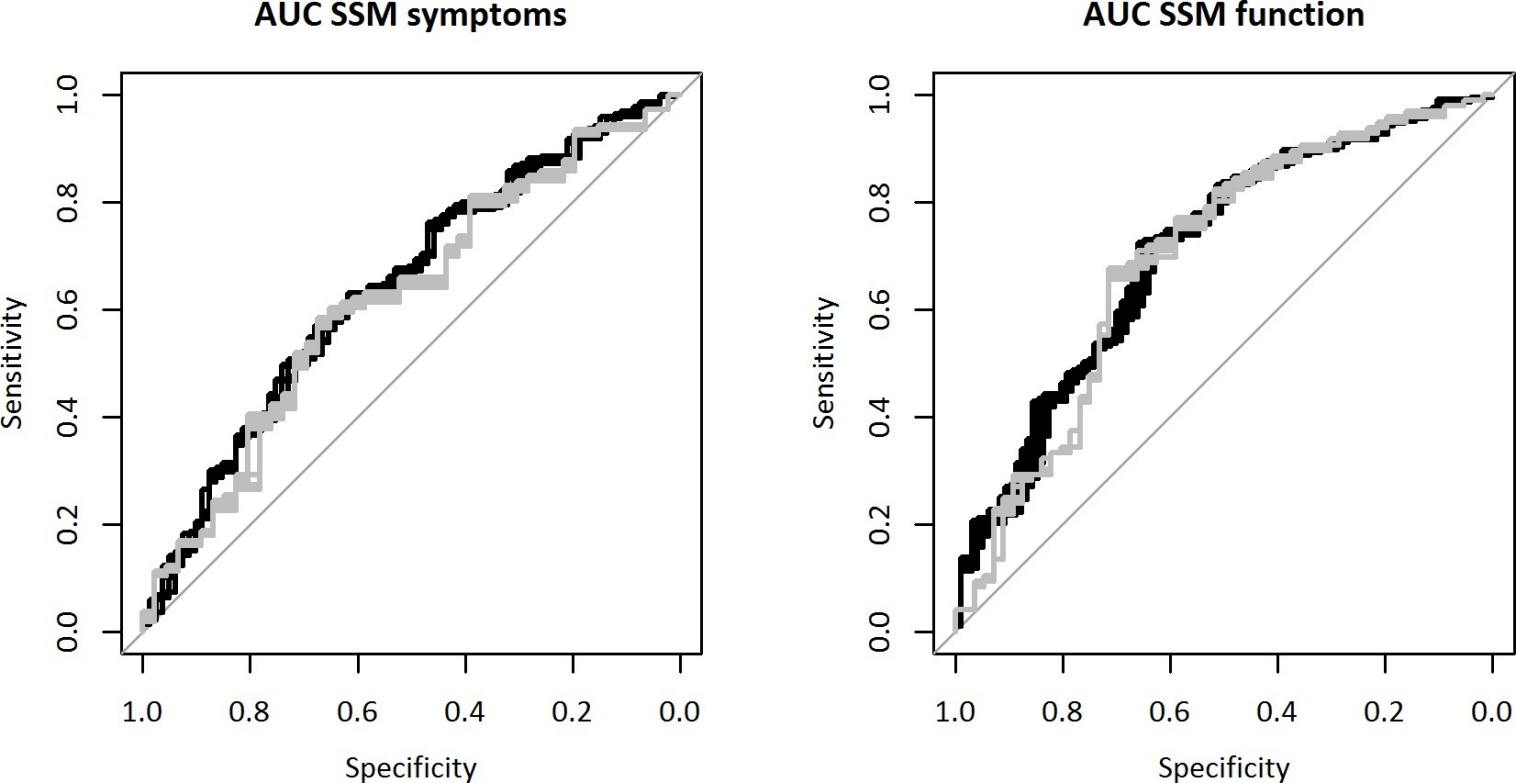
Receiver operating characteristic curves for MCID in SSM symptoms score and SSM function score. · Black lines show results from ten imputed derivation subsamples, grey lines show results from ten imputed validation subsamples. The discriminative ability of the PPF was 0.64 (derivation = black lines) and 0.62 (validation = grey lines) for SSM symptoms score (left panel). The corresponding values were 0.71 and 0.70 for SSM function score as outcome (right panel). MCID = meaningful clinically important difference; SSM = Spinal Stenosis Measure.

The corresponding ROC curves are displayed in Fig 2 by applying the PPF with shrinked coefficients to the ten derivation (black lines) and the ten validation (grey lines) subsamples resulting from the multiple imputation. The left panel shows the MCID in SSM symptoms score, and the right panel shows MCID in SSM function score.

**Fig 3:**
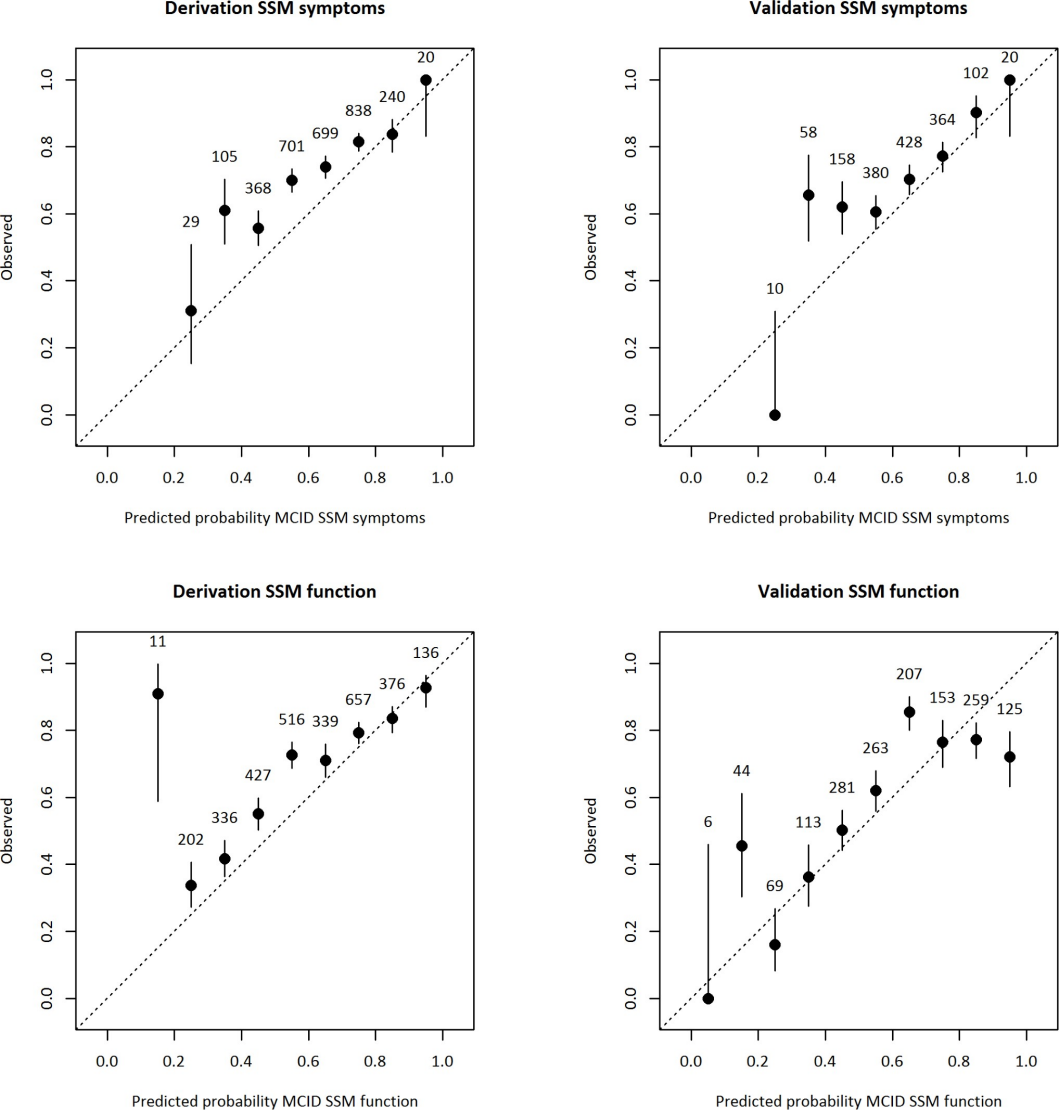
Calibration plots of observed versus predicted probabilities for MCID in SSM symptoms score and SSM function score. The left panel shows results from the derivation subsample, and the right panel shows results from the validation subsample. Overall, there was good calibration of the PPF when probabilities for MCID were displayed against bins of observed probabilities for MCID, shown in upper panels for SSM symptoms score and lower panels for SSM function score. MCID = meaningful clinically important difference; SSM = Spinal Stenosis Measure; MI = multiple imputation.

The calibration plots in Fig 3 show that there was good overall calibration of the PPFs when probabilities for MCID were displayed against bins of observed probabilities for MCID. The upper panels show results for SSM symptoms score, and the lower panels show results for SSM function score.

The information whether fusion was added or not to decompression surgery was entered as an additional variable to the PPF. The resulting AUC of the PPF in the derivation subsample was 0.64 (95% CI 0.57 to 0.71) for the MCID in SSM symptoms score after shrinkage and 0.62 (0.52 to 0.72) in the validation subsample. The corresponding values for the MCID in SSM function score were 0.71 (0.64 to 0.77) and 0.68 (0.59 to 0.77).

### Specific patient scenarios

The estimated probability for MCID after surgery in SSM symptoms score was 81% in the favorable scenario (Scenario 1), and 9% in the unfavorable scenario (Scenario 2). For the MCID in SSM function score, the first scenario resulted in an estimated probability of success of 97%, while it was only 6% in the second scenario.

We demonstrate how these two scenarios lead to the aforementioned probabilities in S1 and S2 Tables. The probabilities of every other constellation of prognostic indicators can be calculated online at www.evimed.ch/PROCESS.

## Discussion

We derived and validated PROCESS in a population of 452 patients with lumbar spinal stenosis in order to estimate the probability of reaching a minimal clinically important difference (MCID) one year after baseline. The discriminative ability and calibration of the PPF for MCID in SSM function score was better than that for SSM symptoms score. Approximately two thirds of the patients benefitted from spinal surgery, however, preoperative prognostic indicators had a large impact on individual outcomes. High baseline pain or functional impairment levels were among the strongest indicators positively associated with MCID in symptoms or function. Depression, low back pain, and previous lumbar surgery were negatively associated. Estimated probabilities of MCID varied, and ranged from 6% to 97%.

The authors of current treatment guidelines identified limited evidence to recommend surgical treatment for patients with lumbar spinal stenosis [35], and did not address the importance of prognostic indicators in the treatment decision. Several prognostic indicators associated with clinically meaningful improvement were identified in a systematic review. These included better reported walking capacity, better self-rated health, and shorter symptom duration [22]. Indicators for an unfavorable outcome after surgery were cardiovascular comorbidity, low back pain, and higher outcome expectations [22]. The majority of the original studies included in the systematic review were of “low quality” and based on small patient samples, likely leading to overly simplified prediction models based on a single or a few prognostic indicators. More recently, several studies identified that a higher degree of baseline disability is associated with increased improvement of functional outcome [20, 36–38], while smoking [36, 39, 40] and psychiatric disease [20, 41] were associated with an unfavorable outcome. Athiviraham et al.[20] reported that higher BMI was associated with less functional improvement, while Pearson et al. [36] reported no difference in function between patients with BMI greater than or equal to 30 and those with a BMI below 30. A few recently published studies considered only a limited patient sample [20, 21, 37, 38] and did not investigate the influence of the indicators on established clinically meaningful improvement [20, 37, 38].

In PROCESS, all available prognostic indicators previously identified were simultaneously included. Given the serious problem of overfitting in the development of PPFs in a large database, we deliberately refrained from an additional selection of parameters collected in the LSOS database, as this would have resulted in optimism regarding the model’s predictive performance in new patients [42]. To address optimism in PROCESS, shrinkage was applied to the regression coefficients and the final models were validated in a random sample of one third of patients previously withheld from the analysis.

The discriminative ability of PROCESS was not altered by the inclusion of the information whether fusion was added or not to decompression surgery.

This study has several strengths. The data were collected prospectively in multiple study centers, and the disease-specific questionnaires SSM symptoms score and SSM function score were used to measure pain and disability. We also applied advanced methodology to obtain a robust PPF using multiple imputation techniques and shrinkage. The performance of the PPF was measured with an unused validation portion of the data set.

A weakness of our study is the fact that the prognostic indicators “coronary heart disease or cardiac insufficiency”, “asthma or COPD”, and “Parkinson’s disease or peripheral neuropathy” happened to have a low prevalence in our data set. Another weakness is that not all risk factors published by Aalto et al. [21, 22] and Athiviraham et al. [20] were collected in the LSOS study: we had no information on preoperative scoliosis (an exclusion criterion in our study when >15°), income, or outcome expectations.

Our method provided clinicians with individualized estimates of success probability with respect to pain and functional improvement that was both easy to understand and simple to communicate to the patient.

Surgical treatment for patients with lumbar spinal stenosis is being performed with increasing frequency [43], leading to higher costs for the health care system. PROCESS is conditional on the individual pattern of preoperatively available prognostic indicators, and may be helpful for clinicians in counselling patients and in guiding the discussion on individual treatment decision in the era of personalized medicine.

## Supporting information

S1 Table Use of the PROCESS prognostic probability function for the MCID in SSM symptoms outcome: Favorable and unfavorable constellation as described in the Methods and Results sections. (DOCX)

S2 Table Use of the PROCESS prognostic probability function for the MCID in SSM function outcome: Favorable and unfavorable constellation as described in the Methods and Results sections. (DOCX)

S1 File STROBE Statement—checklist of items that should be included in reports of observational studies. (DOCX)

S2 File TRIPOD checklist: Prediction model development. (DOCX)
