# Supplementary material for: Prognostic function to estimate the probability of meaningful clinical improvement after surgery - Results of a prospective multicenter observational cohort study on patients with lumbar spinal stenosis
Source: PLoS One. 2018 Nov 8;13(11):e0207126. doi: 10.1371/journal.pone.0207126 (PMC6224088; doi:10.1371/journal.pone.0207126)
Supplement: S1 Table — (DOCX) [file pone.0207126.s001.docx]

**S1 Table.** **Use of the PROCESS prognostic probability function for the MCID in SSM symptoms outcome: favorable and unfavorable constellation as described in the Methods and Results sections.**

|  | | **Favorable constellation** | | **Unfavorable constellation** | |
| --- | --- | --- | --- | --- | --- |
|  | **Shrinked coefficients** | **Prognostic indicators^B^** | **Shrinked coefficents *multiplied with* prognostic indicators** | **Prognostic indicators^A^** | **Shrinked coefficents *multiplied with* prognostic indicators** |
| (Intercept) | -0.565 | 1 | -0.565 | 1 | -0.565 |
| Age ≥75 years | -0.214 | 0 | 0 | 1 | -0.214 |
| Female gender | 0.050 | 0 | 0 | 1 | 0.050 |
| BMI ≥30 kg/m^2^ | -0.207 | 0 | 0 | 1 | -0.207 |
| Current smoker | -0.010 | 0 | 0 | 1 | -0.010 |
| Living alone, or single/divorced/widowed and living in nursing home | -0.083 | 0 | 0 | 1 | -0.083 |
| Compulsory school only | -0.108 | 0 | 0 | 1 | -0.108 |
| Coxarthrosis or gonarthrosis | -0.186 | 0 | 0 | 1 | -0.186 |
| Coronary heart disease or cardiac insufficiency | -0.569 | 0 | 0 | 1 | -0.569 |
| Asthma or COPD | 0.285 | 0 | 0 | 1 | 0.285 |
| Parkinson’s disease or peripheral neuropath | -0.568 | 0 | 0 | 1 | -0.568 |
| Being able to walk only up to 200m | -0.088 | 0 | 0 | 1 | -0.088 |
| Low back pain | -0.205 | 0 | 0 | 1 | -0.205 |
| Duration of symptoms ≥6 months | 0.002 | 0 | 0 | 1 | 0.002 |
| Preoperative analgesic use | -0.033 | 0 | 0 | 1 | -0.033 |
| Previous lumbar surgery | 0.023 | 0 | 0 | 1 | 0.023 |
| More than one decompressed level | -0.125 | 0 | 0 | 1 | -0.125 |
| Diameter of the dural sac (APD) >6 mm or cross sectional area >70 mm^2^ | -0.324 | 0 | 0 | 1 | -0.324 |
| Depression on HADS scale ≥8 | -0.509 | 0 | 0 | 1 | -0.509 |
| Quality of life (EQ5D-3L scale) | 0 | 50 | 0 | 50 | 0 |
| Baseline SSM symptoms | 0.652 | 3.5 | 2.282 | 2 | 1.304 |
| Baseline SSM function | -0.081 | 3.5 | -0.284 | 2 | -0.162 |
| *Sum* |  |  | *1.434* |  | *-2.292* |
| **Inverse logit^B^ of sum** |  |  | **0.81 = 81%** |  | **0.09 = 9%** |

^A^ for binary coefficients: 1 = present, 0 = absent; for continuous coefficients: score

**^B^** inverse logit: exp(x)/(1+exp(x)); x = *Sum*

COPD = chronic obstructive pulmonary disease; HADS = Hospital Anxiety and Depression Scale; SSM = Spinal Stenosis Measure
