# Supplementary material for: Prognostic function to estimate the probability of meaningful clinical improvement after surgery - Results of a prospective multicenter observational cohort study on patients with lumbar spinal stenosis
Source: PLoS One. 2018 Nov 8;13(11):e0207126. doi: 10.1371/journal.pone.0207126 (PMC6224088; doi:10.1371/journal.pone.0207126)
Supplement: S2 Table — (DOCX) [file pone.0207126.s002.docx]

**S2 Table.** **Use of the PROCESS prognostic probability function for the MCID in SSM function outcome: favorable and unfavorable constellation as described in the Methods and Results sections.**

|  | | **Favorable constellation** | | **Unfavorable constellation** | |
| --- | --- | --- | --- | --- | --- |
|  | **Shrinked coefficients** | **Prognostic indicators^A^** | **Shrinked coefficents *multiplied with* prognostic indicators** | **Prognostic indicators^A^** | **Shrinked coefficents *multiplied with* prognostic indicators** |
| (Intercept) | -0.633 | 1 | -0.633 | 1 | -0.633 |
| Age ≥75 years | -0.362 | 0 | 0 | 1 | -0.362 |
| Female gender | -0.001 | 0 | 0 | 1 | -0.001 |
| BMI ≥30 kg/m^2^ | -0.574 | 0 | 0 | 1 | -0.574 |
| Current smoker | 0.280 | 0 | 0 | 1 | 0.280 |
| Living alone, or single/divorced/widowed and living in nursing home | 0.355 | 0 | 0 | 1 | 0.355 |
| Compulsory school only | 0.068 | 0 | 0 | 1 | 0.068 |
| Coxarthrosis or gonarthrosis | -0.427 | 0 | 0 | 1 | -0.427 |
| Coronary heart disease or cardiac insufficiency | -0.445 | 0 | 0 | 1 | -0.445 |
| Asthma or COPD | -0.433 | 0 | 0 | 1 | -0.433 |
| Parkinson’s disease or peripheral neuropath | -0.662 | 0 | 0 | 1 | -0.662 |
| Being able to walk only up to 200m | 0.065 | 0 | 0 | 1 | 0.065 |
| Low back pain | -0.676 | 0 | 0 | 1 | -0.676 |
| Duration of symptoms ≥6 months | -0.028 | 0 | 0 | 1 | -0.028 |
| Preoperative analgesic use | -0.220 | 0 | 0 | 1 | -0.220 |
| Previous lumbar surgery | -0.592 | 0 | 0 | 1 | -0.592 |
| More than one decompressed level | -0.226 | 0 | 0 | 1 | -0.226 |
| Diameter of the dural sac (APD) >6 mm or cross sectional area >70 mm^2^ | -0.343 | 0 | 0 | 1 | -0.343 |
| Depression on HADS scale ≥8 | -0.331 | 0 | 0 | 1 | -0.331 |
| Quality of life (EQ5D-3L scale) | 0.006 | 50 | 0.300 | 50 | 0.300 |
| Baseline SSM symptoms | -0.143 | 3.5 | -0.501 | 2 | -0.286 |
| Baseline SSM function | 1.223 | 3.5 | 4.281 | 2 | 2.446 |
| *Sum* |  |  | *3.447* |  | *-2.725* |
| **Inverse logit^B^ of sum** |  |  | **0.97 = 97%** |  | **0.06 = 6%** |

^A^ for binary coefficients: 1 = present, 0 = absent; for continuous coefficients: score

**^B^** inverse logit: exp(x)/(1+exp(x)); x = *Sum*

COPD = chronic obstructive pulmonary disease; HADS = Hospital Anxiety and Depression Scale; SSM = Spinal Stenosis Measure
